# Gradient system characterization of a 1.5 T MR‐Linac with application to 4D UTE imaging for adaptive MR‐guided radiotherapy of lung cancer

**DOI:** 10.1002/mrm.30505

**Published:** 2025-03-19

**Authors:** Rosie Goodburn, Tom Bruijnen, Bastien Lecoeur, Prashant Nair, Merina Ahmed, Helen Barnes, Uwe Oelfke, Andreas Wetscherek

**Affiliations:** ^1^ Joint Department of Physics Institute of Cancer Research and Royal Marsden NHS Foundation Trust London United Kingdom; ^2^ The Royal Marsden NHS Foundation Trust Sutton United Kingdom; ^3^ Department of Radiotherapy University Medical Center Utrecht Utrecht The Netherlands; ^4^ Department of Computing Imperial College London London United Kingdom

**Keywords:** GIRF, GSTF, lung cancer, MR‐Linac, UTE

## Abstract

**Purpose:**

To measure the gradient system transfer function (GSTF) of an MR‐Linac (Elekta Unity, Stockholm, Sweden) using an accessible phantom‐based method and to apply trajectory corrections for UTE image reconstruction in the context of MR‐guided radiotherapy of lung cancer.

**Methods:**

The first‐order GSTF of a 1.5 T, split gradient Elekta Unity MR‐Linac was measured using a thin‐slice technique to characterize gradient system imperfections for each physical gradient axis (X, Y, Z). Repeatability measurements of the GSTF were performed 48 h apart. The GSTF was applied to trajectory correction in multi‐echo UTE image reconstruction (TEs = 0.176, 1.85, 3.52 ms) to allow for UTE‐Dixon inputs in the generation of synthetic CT. Images were acquired in an anthropomorphic phantom and in two free‐breathing lung cancer patients. For patient scans, respiratory‐correlated 4D‐MR images were reconstructed using self‐navigation and an iterative compressed‐sensing algorithm.

**Results:**

The GSTF magnitude was similar across the X/Y/Z axes up to ˜6 kHz. The GSTF phase was similar between the X/Y/Z components up to ˜3 kHz. Repeatability measurements demonstrated minimal variations corresponding to a system delay difference of 0.06 μs. Corrected UTE trajectory spokes are shifted approximately 1 m^−1^ compared to the nominal *k*‐space location. Corrected phantom and patient UTE images exhibited improved signal uniformity and contrast and reduced halo and signal loss artifacts. Trajectory correction for the later TE images did not improve overall image quality.

**Conclusion:**

The proposed GSTF measurement method using standard MR‐Linac hardware enables successful trajectory correction in UTE imaging reconstruction, with applications to lung synthetic CT generation for MR‐guided radiotherapy.

## 1 INTRODUCTION

Synthetic CT (synCT) techniques for adaptive MR‐guided radiotherapy (MRgRT) of lung cancer can benefit from incorporating UTE imaging, which provides contrast in short‐$\;T_{2}^{*}$ lung tissues.^1^, ^2^, ^3^, ^4^ In particular, this imaging could allow for accurate assignment of lung CT numbers in patients with emphysema, which otherwise might be indistinguishable from normal lung parenchyma.^5^ UTE techniques achieve sub‐millisecond nominal TEs through non‐Cartesian, center‐out *k*‐space trajectories, which sample the signal during the ramping up of the readout gradients.^6^ However, actual waveforms are distorted from their ideal shapes due to “field settling” effects arising from gradient‐chain imperfections, which include limited coil and amplifier bandwidths, mechanical vibrations, a finite accuracy of the timing calibration, and eddy currents.^7^, ^8^ Without trajectory corrections, UTE images can suffer significant degradation due to inaccurate k‐data mapping, particularly near the center of *k*‐space.

UTE nominal trajectories, therefore, are typically corrected for field‐settling behavior, with measurement‐based corrections proving more accurate than model‐based approaches.^8^, ^9^ A generalized correction may be achieved by estimating or measuring a gradient system transfer function (GSTF), which enables correction of any arbitrary gradient waveform, assuming linear time‐invariant behavior.^8^, ^10^, ^11^ The GSTF is determined by comparing ideal, nominal waveforms—typically triangular pulses or chirps—to measured, output waveforms. These output waveforms can be measured with specialized NMR field probes^8^, ^12^ or by using scanner hardware in combination with simple phantoms.^13^, ^14^, ^15^

Field‐settling effects characterized with GSTFs are effectively modeled as a combination of exponential decays with various time constants, with the induced magnetic fields characterized as polynomials in 3D space.^7^, ^16^ Zeroth‐order (*B*_*0*_) components cause brief, spatially uniform field offsets.^17^ First‐order (*linear*) components, which generate first‐order magnetic fields that vary linearly in space, mimic the imaging gradient fields and distort *k*‐space trajectories. To mitigate linear effects, active shielding of the gradient coils and preemphasis pulses are used to counteract anticipated distortions. However, these methods are generally not fine‐tuned enough to fully remove the short‐time constant effects that impact UTE imaging. Higher‐order field settling components, which produce spatially nonlinear magnetic fields, are typically insignificant relative to zeroth‐ and first‐order effects in UTE imaging.^18^

The aim of this work is to present a simple phantom‐based technique for characterizing the GSTF of a split gradient, 1.5 T MR‐linear accelerator (Linac) (Elekta Unity, Stockholm, Sweden) with application to UTE imaging for adaptive MRgRT of lung cancer. To the authors' knowledge, the GSTF measurement method used in this work has not been previously demonstrated for UTE lung imaging on either diagnostic or MR‐Linac systems. The GSTF is used to correct trajectories and demonstrated for reconstruction of 4D multi‐echo UTE lung MRI in a phantom and in vivo for patients imaged on the MR‐Linac (Elekta Unity) with a sequence designed to provide UTE‐Dixon images as an input to synCT generation for adaptive MRgRT.

## 2 METHODS

The GSTF measurement in this work is based on the relation:

(1) $$\phi_{r}\left(t\right)=2\pi \;D_{r}\;k_{r}\left(t\right)=\gamma \;D_{r}\;\int_{0}^{t}g_{r}\left(t^{\prime }\right)\;dt^{\prime },$$

where r is the physical gradient axis (X/Y/Z), $\phi_{r}\left(t\right)$ is the relative phase of the measured signal at a distance $D_{r}$ from the isocenter of the system, $k_{r}\left(t\right)$ is the *k*‐space trajectory, and $g_{r}\left(t\right)$ is the imaging gradient.^15^ Therefore by measuring $\phi_{r}\left(t\right)$, the true, measured waveform, $g_{r,\text{meas}}\left(t\right)$ can be calculated and compared with the nominal waveform, $g_{r,\mathrm{nom}}\left(t\right)$. By modeling the gradient system as linear time‐invariant, we take $g_{r,\text{meas}}\left(t\right)$ to be equivalent to the convolution of the nominal gradient waveform with a gradient impulse response function ("GIRF"), $h_{r}\left(t\right)$, (the Fourier transform of the GSTF, $H_{r}\left(f\right)$):

(2) $$g_{r,\text{meas}}\left(t\right)=\int_{-\infty }^{+\infty }g_{r,\mathrm{nom}}\left(t\right)\;h_{r}^{1}\left(t-\tau \right)\;d\tau .$$

We expect zeroth‐order effects to be insignificant in UTE applications because the central portion of *k‐*space is sampled early such that the impact of additional signal phase is minimal.^19^ Because the primary aim of this work is correcting UTE imaging, only the first‐order GSTF ($H_{r}^{1}\left(f\right)$), which captures the linear effects of gradient imperfections, is measured here. To ensure coverage over the desired frequency spectrum, $H_{r}^{1}\left(f\right)$ can be estimated by applying a range of triangular pulses, each targeting a different set of spectral frequencies:^20^

(3) $$H_{r}^{1}\left(f\right)=\frac{\sum_{i}G_{r,\mathrm{nom}}^{i}\left(f\right)\;G_{r,\text{meas}}^{i}\left(f\right)}{\sum_{i}{\left|G_{r,\mathrm{nom}}^{i}\left(f\right)\right|}^{2}},$$

where the index i represents a given triangular waveform. Here, $G_{r,\mathrm{nom}}^{i}\left(f\right)$ and $G_{r,\text{meas}}^{i}\left(f\right)$ represent the Fourier transforms of the nominal and measured gradient waveforms, respectively, which are derived using Equation (1), with corrections applied to isolate linear effects (see below).

### 2.1 GSTF measurement

**FIGURE 1:**
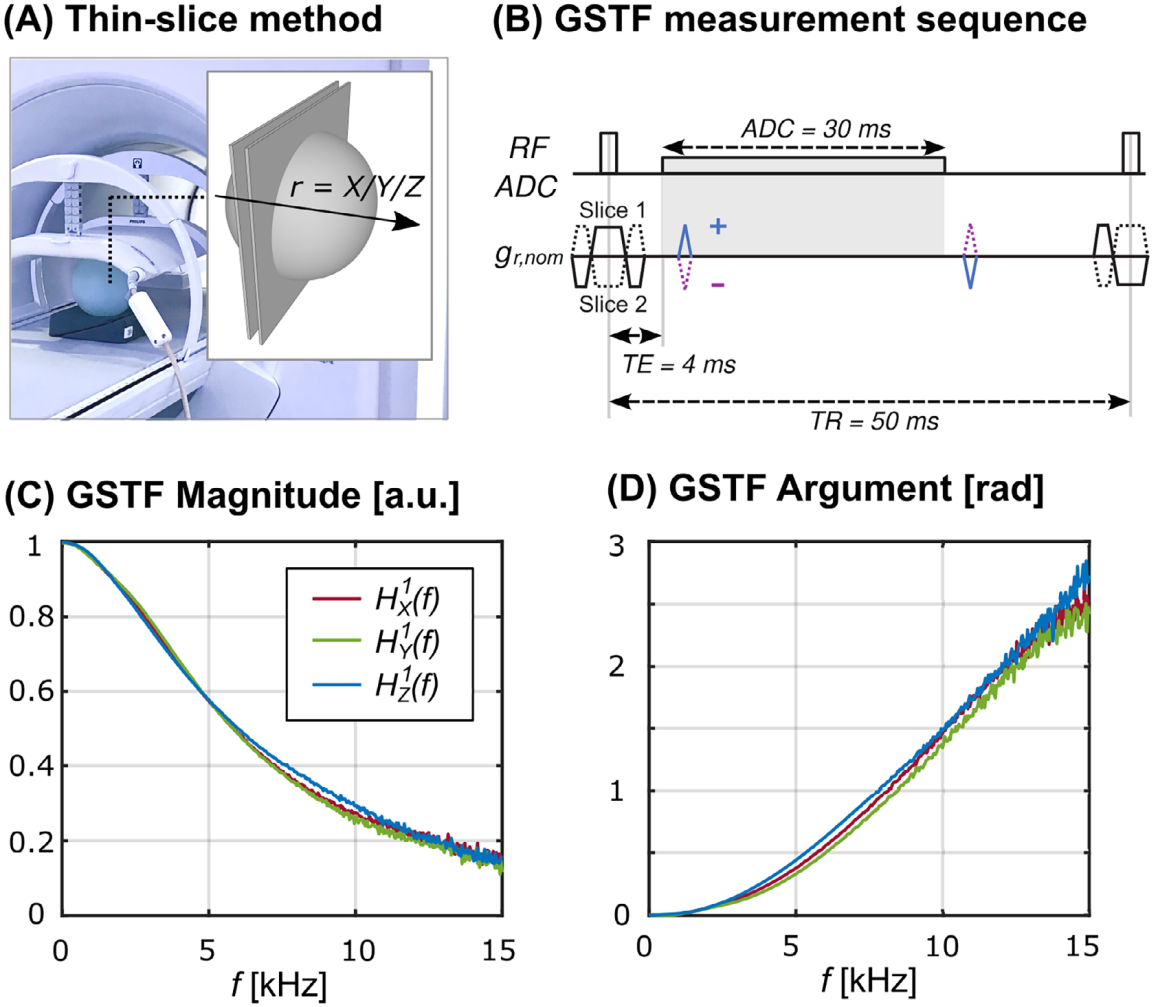
GSTF measurement with a thin‐slice method. (A) Schematic diagram of the thin‐slice method, which uses two parallel slices per gradient direction. For each physical gradient axis, two thin slices were positioned symmetrically at 20 mm from the isocenter, perpendicular to the gradient axis being measured. (B) Pulse sequence diagram for the GSTF measurement, showing triangular gradient waveforms (blue, purple) at 21 different amplitudes, ranging from 4 to 15 mT/m, with a maximum slew rate of 65 mT/m/ms. (C, D) Measured spectra of the first‐order GSTF for the three gradient directions, displaying the magnitude and phase (argument) of the complex‐valued GSTF over the range of 0 to 15 kHz. GSTF, gradient system transfer function.

Figure 1 illustrates the thin‐slice method^15^, ^17^, ^20^, ^21^ used to characterize $H_{r}^{1}\left(f\right)$ for the 1.5 T MR‐Linac system (Elekta Unity) at the Royal Marsden Hospital, London, UK. A 15‐cm diameter spherical phantom was positioned at isocenter (with tolerance of ±10 mm in any direction) with four‐channel anterior and four‐channel posterior coils used for signal reception. During acquisition, triangular gradient pulses were applied in two 3‐mm thick slices symmetrically placed 20 mm off‐isocenter. To achieve sufficient spectral density, the GSTF measurement sequence (Figure 1B) was performed with 21 different amplitudes of triangular gradients (blue, purple) ranging from 4 to 15 mT/m, with a maximum slew rate of 65 mT/m/ms. To allow for the removal of static field inhomogeneity effects, triangular pulses cycled between positive and negative polarity. A separate measurement was run for each GSTF component (X/Y/Z). The total sampling time of the readout for this measurement was 30 ms, with a sampling frequency of 1.67 MHz, corresponding to a dwell time of 0.6 μs. Other parameters: TR = 50 ms, number of averages = 100, scan time = 630 s per gradient axis.

Offline processing was performed in MatLab 2020b (MathWorks, Natick, MA), using a PC with an 18‐core Intel Core CPU (Intel, Santa Clara, CA) and 256 GB of Crucial Pro RAM (Micron Technology, Boise, ID). Here, the complex signal data were compressed using singular‐value decomposition across the coil channels.^22^ The *k*‐space data were phase‐aligned by setting the phase of the first sample to zero. Complex averaging was performed before calculating the complex phase and performing phase unwrapping. Phase contributions from static off‐resonances and concomitant gradients were removed by subtracting the negative‐pulse measurements from the positive‐pulse measurements. Subsequently, zeroth‐order effects were found and removed by subtracting measurements from the two slices. The residual phase accumulation for each of the 21 triangular pulses was attributed to first‐order effects, and the GSTF was calculated following Equation 3.^8^ Repeatability measurements were made 48 h apart with the same Linac‐gantry angle (0°), and at least 1 h after other scanning. Additional GSTF measurements were also acquired for different gantry angles set at 0, 90, 180, and 270°.

### 2.2 UTE imaging acquisition

**FIGURE 2:**
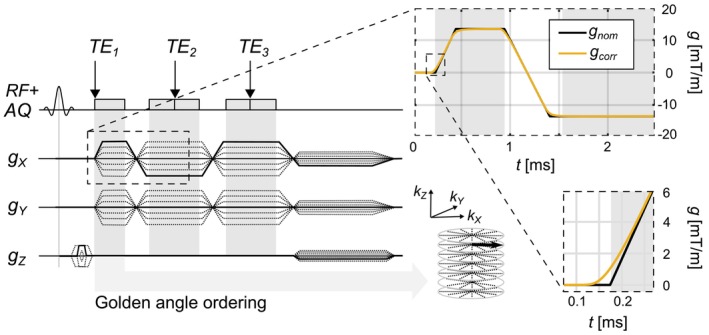
Pulse sequence diagram for the 3D gradient‐echo multi‐echo UTE sequence. A selective RF pulse was used to excite a ≈ 400‐mm length slab covering the thorax. Variable‐duration slice encoding was employed for *k*‐space encoding along *k*_z_. A stack‐of‐stars *k*‐space sampling scheme with golden‐angle spoke ordering and centric‐ordered *k*_z_ slice encoding was used. The nominal and corrected gradient waveforms are displayed, highlighting a substantial correction at the TE of the UTE signal, where this correction is expected to have the greatest impact on image quality.

Figure 2 illustrates the multi‐echo UTE pulse sequence diagram as well as the effect of GSTF correction on the UTE gradient waveform. A selective RF pulse is used to excite a slab covering the thorax, with variable‐duration slice encoding employed to partition *k*‐space.^23^ A stack‐of‐stars sampling scheme is used with golden‐angle (GA) spoke ordering and centric‐ordered slice encoding. The nominal and corrected gradient waveforms are displayed, highlighting a substantial correction at the TE of the UTE signal, where this correction is expected to have the greatest impact on image quality.

All imaging was performed on the 1.5 T MR‐Linac (Elekta Unity) system at our site, using four‐channel anterior and four‐channel posterior phased‐array coils (Philiips, Best, The Netherlands) for signal reception. To evaluate the effectiveness of GSTF correction, imaging was first conducted on an anthropomorphic phantom (3D Abdominal, CIRS, VA). To assess clinical imaging, two patient volunteers who underwent lung cancer treatment were scanned in the supine position with arms up using radiotherapy lung boards for positioning. Patients consented to participate in the PRIMER study (NCT02973828), approved under IRAS: 208449 and REC: 17/LO/0907. A volumetric T_1_‐weighted, gradient‐echo acquisition with slab‐selective excitation and a stack‐of‐stars trajectory was used. Detailed sequence parameters can be found in Table 1. GA spoke ordering was used to allow for binning into dynamic frames,^24^ where each projection angle was acquired in one block along *k*_z_ with centric‐ordered slice encoding. A software patch, generated with the pulse programming environment (Philips Paradise release 5.7.1), was applied at scanning to enable GA sampling with a UTE trajectory.

**TABLE 1:** Parameter settings of the 3D gradient echo (GRE multi‐echo UTE sequence.

Acquisition voxel size (mm^3^)	1.5 × 1.5 × 5.0
FOV (mm^3^)	500 × 500 × 400
Slice orientation	Axial
TEs (ms)	0.176, 1.849, 3.521
TR (ms)	8.29
Bandwidth (Hz/pixel)	865
Flip angle (°)	10
*k*_z_ partitions	103
Slice oversample factor	1.288
Duration (s)	599.9
Spokes per partition	664
Samples per spoke	392 (TE_1_), 664 (TE_2,3_)
Readout oversampling	2

Abbreviation: GRE, gradient echo.

### 2.3 Trajectory correction

Trajectories ($k_{x}$, $k_{y}$) were corrected by application of the respective GSTF component ($H_{X}^{1}\left(f\right)$, $H_{Y}^{1}\left(f\right)$) with the imported nominal (X, Y) gradient waveforms ($g_{X,\mathrm{nom}}\left(t\right)$, $g_{Y,\mathrm{nom}}\left(t\right)$). Nominal waveforms were prepared for trajectory correction by resampling to a 0.1 μs grid, followed by zero‐padding (±5 ms) and transformation to spectral frequency with a Fourier transform. To reduce noise at higher frequencies of the GSTF, a Gaussian filter (SD 0.6 kHz) was applied to the GSTF at frequencies larger in magnitude than ±6 kHz. The GSTF was multiplied by a Tukey filter (taper of 0.1) to avoid ringing in the corrected waveform, and the processed GSTF was resampled to match the raster of the spectral waveform. Following multiplication in frequency space, corrected gradients ($g_{X,\text{corr}}\left(t\right)$, $g_{Y,\text{corr}}\left(t\right)$) were generated by transforming back to the time domain. Finally, corrected trajectories were calculated using the relation in Equation (1).

### 2.4 Phantom images: offline reconstruction

All image reconstruction was performed in MatLab 2020b (MathWorks, Natick, MA) using a PC with an 18‐core Intel Core CPU and 256 GB of RAM.

To enable slice‐by‐slice reconstruction, an inverse fast Fourier transform along the Cartesian *k*_z_ dimension was applied. Coil sensitivity maps were generated from the temporally averaged UTE data using the eigenvector‐based iterative self‐consistent parallel imaging reconstruction^25^ implementation of the Berkeley Advanced Reconstruction Toolbox (v0.8.00).^26^ Motion‐averaged images with a resolution of 1.51 × 1.51 × 15.00 mm were reconstructed for each slice and TE by applying the coil sensitivity maps and an inverse 2D nonuniform fast Fourier transform (NUFFT)^27^ from the Berkeley Advanced Reconstruction Toolbox.

### 2.5 Patient images: self‐navigation and binning

Respiratory self‐gating was based on the signal from the *k*‐space center. For each spoke angle, the magnitude of the signal from the nine sample points closest to the center of *k*‐space was calculated separately for eight coils and 103 *k*_z_ partitions. A background subtraction, using the moving average of neighboring angles, was applied to the signal.^28^, ^29^ Following this correction, a quality measure for the respiratory signal was evaluated for each receive coil using the method described by Grimm et al.^30^ The coils with the poorest signal quality—comprising half of the total—were excluded from further processing.

Principal component analysis was performed across the remaining coils. The first principal component was selected as surrogate of the respiratory phase for each spoke angle. If 4D‐MRI reconstructed from this surrogate showed minimal respiratory motion at the top of the diaphragm from visual inspection, the second component was used instead. The resulting respiratory signal was then used to sort each spoke angle into one or two of five motion states based on respiratory phase, ensuring an equal number of time points in each bin. A 50% bin overlap was applied, allowing each spoke to contribute to two adjacent respiratory bins, except for those in the extreme bins, which corresponded to maximum inhalation and exhalation. A TR of 8.61 ms and 103 *k*_z_ partitions resulted in a motion sampling resolution of 0.89 s. Because the principal component could have a positive or negative sign, a visual inspection of the resulting 4D MRI was performed to ensure that the exhalation state was correctly identified, and the sign was reversed in cases that it was switched. One way to automate this step would be correlation with respiratory signals that are directly related to the spatial location, such as the principal component analysis‐based approach used in the extra‐dimensional golden‐angle radial sparse parallel imaging work.^31^

### 2.6 Patient images: offline reconstruction

Following transformation of the respiratory‐binned *k*‐space data along *k*_z_, respiratory‐resolved images were reconstructed by iteratively solving the optimization problem:

(4) $$\underset{d}{\arg\;\min}{\left\|F\left(d,C\right)-y\right\|}_{2}^{2}+\lambda {\left\|S\left(d\right)\right\|}_{1,}$$

with d = dynamic images (*N*_*x*_ *‐N*_*y*_ *‐N*_bin_), F = multi‐coil NUFFT operator,^27^ C = coil sensitivity maps (*N*_*x*_ *‐N*_*y*_ *‐N*_coil_), y = multi‐coil *k*‐space data (*N*_read_ *–N*_proj_ *–N*_coil_ *–N*_bin_), λ = regularization parameter, and S = sparsifying transform. This reconstruction was performed using code adapted from the extra‐dimensional golden‐angle radial sparse parallel imaging method.^31^, ^32^

A conjugate gradient descent algorithm (15 iterations) with line search was used to solve (Equation 4).^31^, ^33^ The iterative process involved the application of type 2 and adjoint NUFFT operators,^27^ incorporating GSTF‐corrected trajectories, density compensation,^34^, ^35^ and coil sensitivity maps (generated using eigenvector‐based iterative self‐consistent parallel imaging reconstruction^25^, ^26^). The sparsifying transform, S, applied a “total difference” penalty along the temporal dimension, which acts as a surrogate for a total variation regularization.^31^, ^32^ The regularization parameter λ was set to 5% of the maximum value of the noniterative NUFFT‐reconstructed image volume.^32^ For each patient, 72 slices (5‐mm thick) were reconstructed, with an in‐plane matrix size of 332 × 332 and a 500 × 500 mm^2^ FOV (1.51 × 1.51 mm resolution).

### 2.7 Image quality assessment

Image quality was assessed visually on phantom images, focusing on signal uniformity, contrast, and the presence of artifacts such as halo effects and signal loss at boundaries. A quantitative measure of blurring was made by plotting a line profile through a bright edge in a central slice of the phantom images and fitting a Gaussian to the derivative to estimate the line spread function (LSF) and the FWHM value of the LSF.

## 3 RESULTS

### 3.1 GSTF measurement

Figure 1C,D display the measured GSTFs, with a spectral resolution of 33 Hz. The magnitude of each GSTF component follows similar patterns up to 6 kHz. Beyond this point, the X and Y gradients show slightly lower transfer ratios compared to the Z gradient, suggesting anisotropic linear effects (e.g., linear eddy currents). The phase response of the GSTF also reveals minor variations at frequencies above approximately 3 kHz across different gradient axes, indicating axis‐specific gradient delays. Whereas noise levels increase at higher frequencies, they do not significantly affect the measurements up to 15 kHz.

### 3.2 GSTF repeatability

**FIGURE 3:**
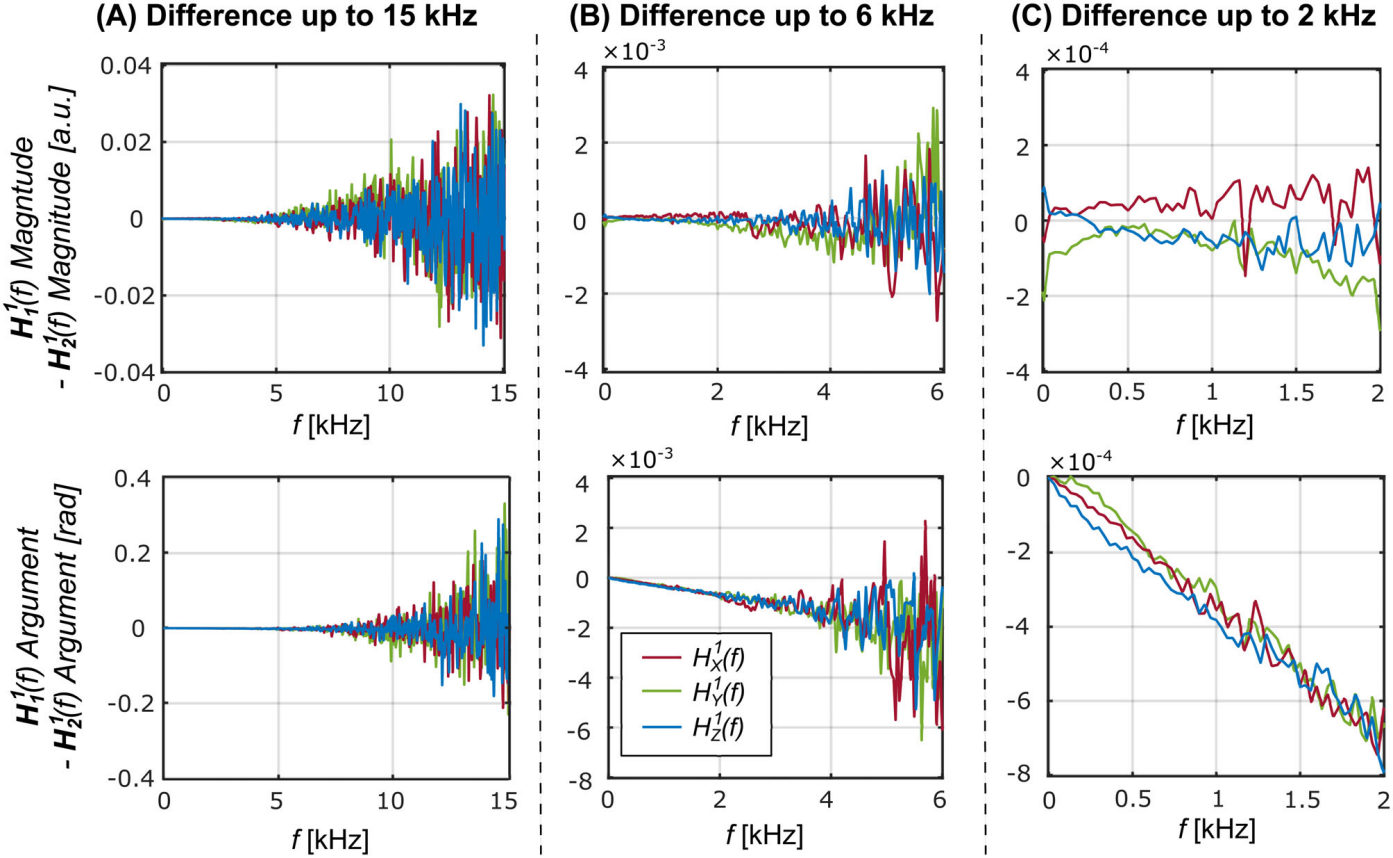
Repeatability of GSTF measurements. The differences in magnitude and phase (argument) between two first‐order GSTF measurements for each physical gradient axis taken 48 h apart, up to 15 kHz (A), 6 kHz (B), and 2 kHz (C). (A) In the 15 kHz range, differences reach <±15% of the mean for the GSTF magnitude and <±10% of the mean for the phase. (B) In the 6 kHz range, magnitude differences are <±1% of the mean. A linear trend in phase deviations is apparent in (C), corresponding to a timing discrepancy of 0.1 μs in the prescribed delay.

Figure 3 illustrates the differences between two first‐order GSTF measurements taken 48 h apart. In Figure 3A (up to 15 kHz), these differences increase with frequency, reaching ±0.03 (<±15% of the mean) for the GSTF magnitude and ± 0.3 rad (<±10% of the mean) for the phase. In Figure 3B (up to 6 kHz), the magnitude differences are within ±3 × 110^−3^, representing <±1% of the mean. In Figure 3C (up to 2 kHz), the maximum differences in magnitude are ±2 × 110^−4^, or <±0.1% of the mean. The phase differences below 2 kHz reach up to 8 × 110^−4^ rad, which appears to represent a linear trend in phase deviations rather than being primarily caused by spectral noise. This corresponds to a timing discrepancy of 0.06 μs in the prescribed group delay close to direct current (zero frequency).^8^, ^36^

Additional measurements were compared for different gantry angles (set at 0, 90, 180, and 270°) during a single scanning session. Comparing all GSTFs measured at each gantry angle, the maximum differences are comparable to the repeatability results. The maximum magnitude difference below 2 kHz was ±7 × 110^−4^, which is <±0.1% of the mean. The greatest phase differences below 2 kHz correspond to a timing discrepancy of the DC group delay of 0.03 μs.

### 3.3 Trajectories and phantom images

**FIGURE 4:**
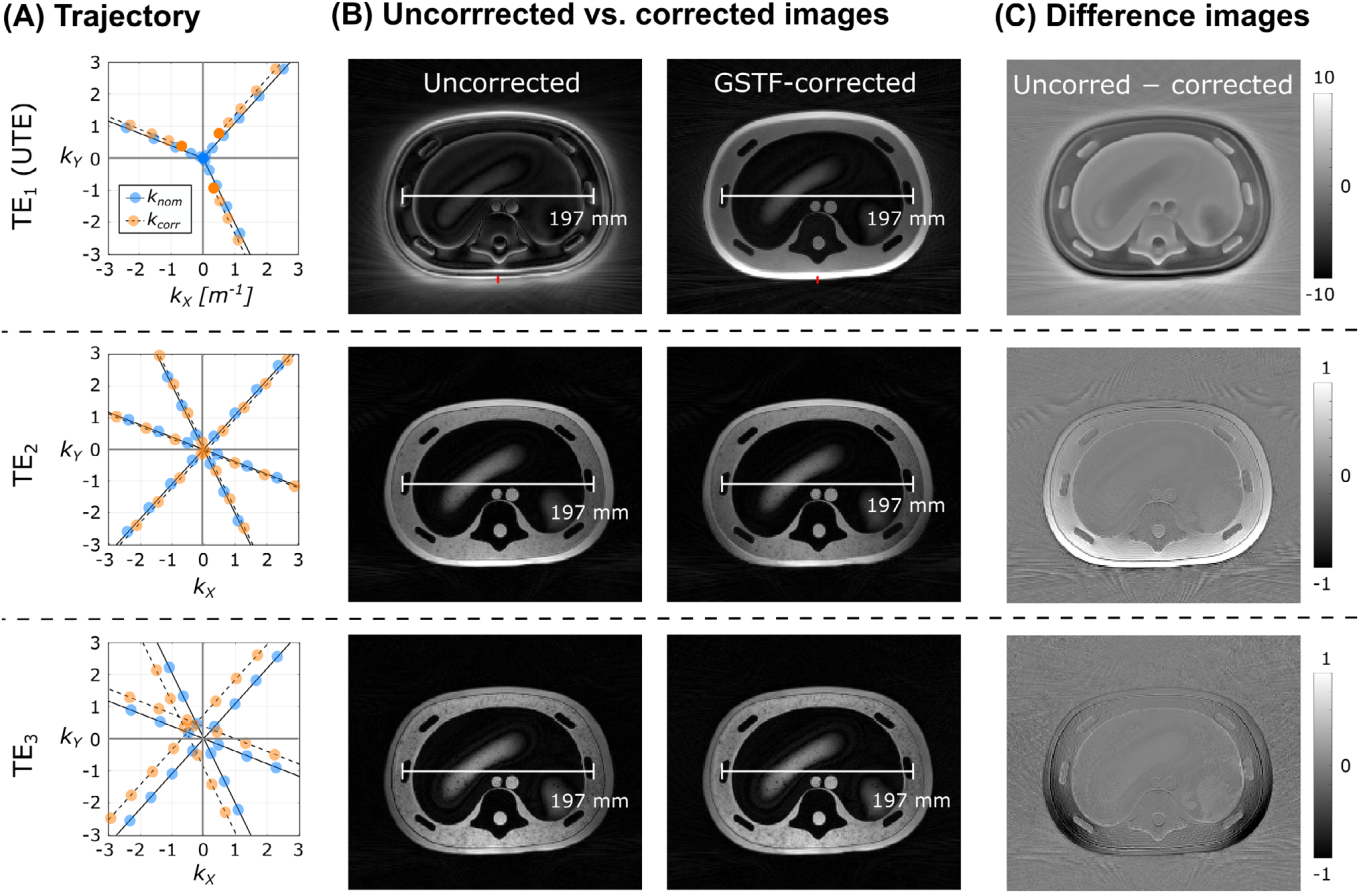
Trajectory correction and its impact on image reconstruction. (A) Comparison of nominal (*k*_nom_, blue) and corrected (*k*_corr_, orange) trajectory locations for the first three spokes in a central 6 × 6 m^−2^ region of *k*‐space. Colored circles indicate every fifth position along the UTE trajectory. (B) Multi‐echo UTE acquisition of an abdominal phantom, reconstructed without as well as with GSTF correction. Distance measurements are provided to illustrate the geometric consistency between TEs. FWHM values of the line spread functions found from the UTE images (line profile shown in red) are 4.1 mm versus 3.0 mm for uncorrected versus corrected images, respectively. (C) Difference images are shown. Note that UTE difference images are displayed with windowing one order of magnitude larger than for the other TEs.

Figure 4A illustrates the *k*‐space trajectory through the central 6 × 16 m^−2^ area for the first three spokes. In the corrected UTE trajectory, each readout spoke begins approximately 1 m^−1^ from the center of *k*‐space, rather than precisely at 0. The *k*‐space spacing is tighter near the center of the UTE trajectory. For the second TE (TE_2_) trajectory, small shifts (˜0.5 m^−1^) are observed parallel to the spokes, whereas for the third TE (TE_3_) trajectory, the shifts are seen perpendicular to the spoke direction. These apparent shifts likely result from the smoothing effect of the GSTF at the corners of the corrected waveform, where the ratio between the X and Y gradient strengths can differ from that of the uncorrected waveforms and where the gradients are assumed to be ideal.

In Figure 4B, phantom images generated using the multi‐echo UTE sequence are shown for a central slice of the abdominal phantom without as well as with GSTF correction. Distance measurements indicate consistent relative scaling between uncorrected and corrected images as well as across different TEs. The corrected UTE images show a clear improvement in image quality, with reduced streaking artifacts in air, boundaries lacking halo and signal loss effects, and improved uniformity and contrast. Measured LSFs of the uncorrected versus corrected UTE images have FWHMs of 4.1 versus 3.0 mm, respectively. Although the UTE images appear more blurred than the other TE images, this is likely due to a different point spread function caused by half‐spoke sampling.^37^ Figure 4C displays the difference images (uncorrected minus corrected). The only notable effect of GSTF correction on the second and third TE images is the change in location of shading artifacts, where these shading artifacts are not present in UTE images.

### 3.4 Patient images

**FIGURE 5:**
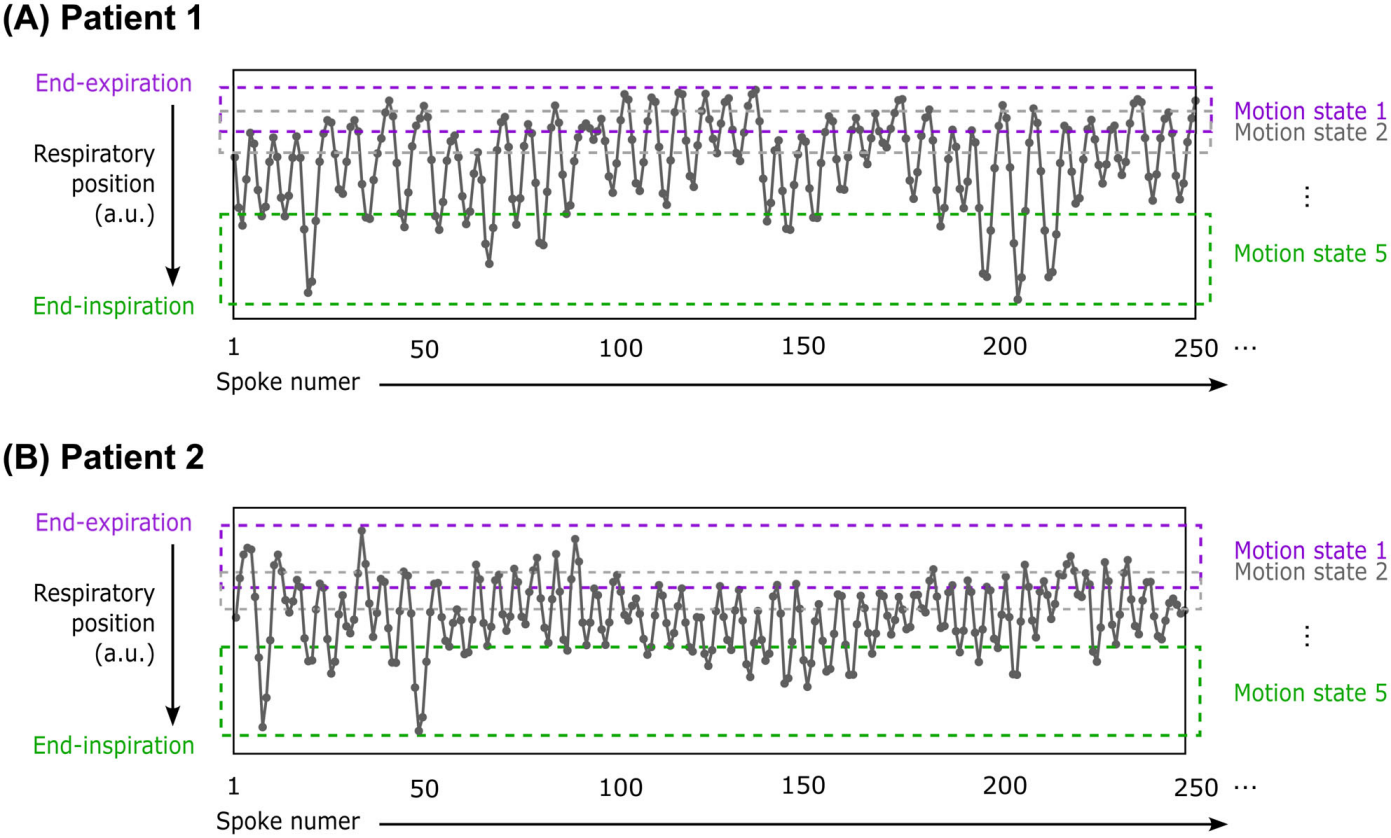
Motion signals and bins generated from two lung cancer patient volunteers. The self‐navigation signal generated from the UTE data for (A) patient 1 and (B) patient 2. The respiratory signal is used to sort each spoke number into one or two of five motion states by allocating an equal number of time points to each bin. A 50% bin overlap is used. The first (end‐inspiration), second, and fifth (end‐expiration) respiratory phases are indicated.

**FIGURE 6:**
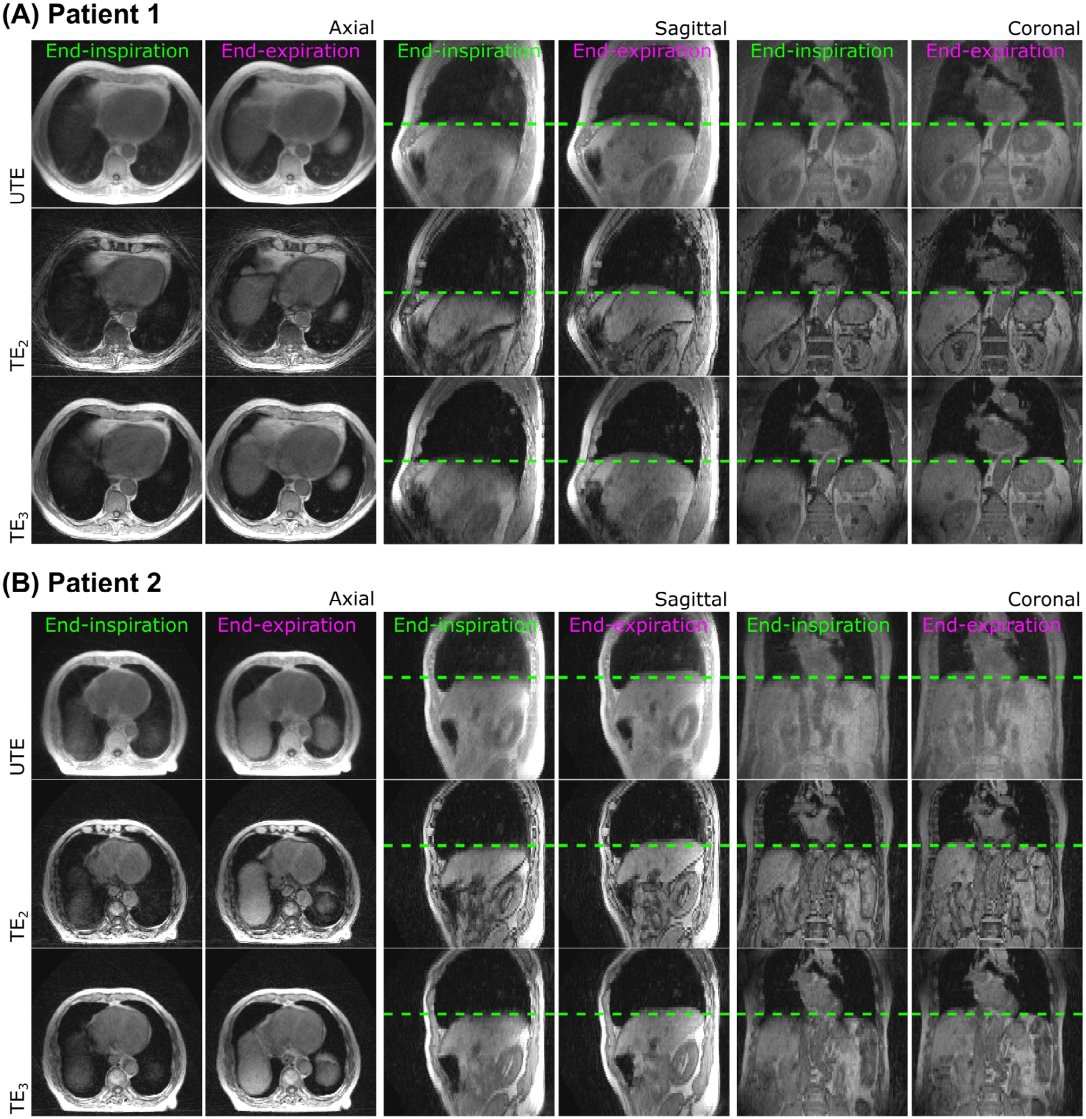
Respiratory‐resolved, GSTF‐corrected images reconstructed from a multi‐echo‐UTE scan of two lung cancer patient volunteers. The GSTF‐corrected images are shown for the first (end‐inspiration) and fifth (end‐expiration) respiratory phases at three TEs (UTE, TE_1_ = 0.18 ms; TE_2_ = 1.8 ms; TE_3_ = 3.5 ms) acquired during free breathing on an MR‐Linac (Elekta Unity, Stockholm, Sweden) for (A) patient 1 and (B) patient 2. The same window and level settings were applied across all images. Dashed green lines indicate the diaphragm's position at end‐inspiration.

Total reconstruction times were 3033 s (˜50 min) per patient. Figure 5 illustrates the self‐navigation signal generated from the UTE data for two representative lung cancer patients and Figure 6 shows the respiratory‐resolved (end‐inspiration and end‐expiration) GSTF‐corrected images. The motion of the diaphragm is highlighted by the dashed green lines in the sagittal view. In these two examples, the top of the diaphragm moves by approximately 1.5 cm and 1.0 cm, respectively. In the UTE images, lung regions appear brighter relative to background air when compared to non‐UTE images, suggesting improved sensitivity to lung density to distinguish between any regions of emphysema and healthy lung for accurate synthetic CT generation. Here, in vivo images appear to have comparable image quality to the equivalent corrected phantom images (Figure 4). Similar to the phantom scans, shading artifacts appear in the second and third TE images with slice‐location dependence. Difference images between uncorrected and corrected images are found in Figure S1.

## 4 DISCUSSION

Incorporating synCT for adaptive MRgRT of lung cancer offers significant benefits by enabling more accurate dose calculations from up‐to‐date images.^5^ However, while vendor‐provided synCT solutions are available for relatively static, homogeneous regions like the brain and pelvis, these solutions have not yet been adapted for the lungs. This gap is also reflected in the research literature.^38^, ^39^ Poor visibility of lung tissue, heterogeneity of the thoracic region, and the effects of respiratory motion further complicate lung synCT generation. Few published methods address the complexities of this anatomy,^2^, ^40^, ^41^ and even fewer are 4D‐ or respiratory‐ resolved.^5^, ^42^, ^43^ As a result, methods that facilitate UTE reconstruction are crucial for advancing the development of thoracic synCT for MRgRT.

The GSTF of a 1.5 T, Elekta Unity MR‐Linac was measured and applied to 4D UTE image reconstruction to support the development of methods for MRgRT of lung cancer, namely UTE and/or UTE‐Dixon lung imaging for the generation of synCT.^5^ The Elekta Unity system uses a split gradient coil design, where the treatment beam is delivered through the gap that separates the two halves.^44^ The Linac components are housed on a large ring gantry that rotates in the axial plane. It is reasonable to expect that this split gradient design is more susceptible to higher‐order eddy currents and heating effects than a comparable diagnostic system, which is why the gradient amplitude is currently capped at 15 mT/m. Bruijnen et al. found that zeroth‐ and first‐order GSTFs were comparable between an Elekta Unity MR‐Linac and Philips diagnostic scanner.^45^ GSTFs of higher spatial order have not yet been measured on the Elekta Unity system. In addition, it is reasonable to ask whether the angle of the gantry affects the measured GSTF. This was briefly investigated in this work, with results indicating minimal differences when compared to other repeatability measurements. Moreover, imaging for treatment planning on the MR‐Linac may be carried out with the gantry at a set position because it is run before the treatment is delivered.

The first‐order GSTF, which characterizes the field settling behavior that impacts trajectories, was measured using a simple phantom with a thin‐slice approach and triangular pulses.^20^ The GSTF was used to correct non‐Cartesian trajectories for 3D and 4D multi‐echo image reconstruction of an anthropomorphic phantom and two lung cancer patients. Corrected images were compared to images reconstructed using the ideal, nominal trajectory. Repeatability measurements of the GSTF showed minimal variations, corresponding to a delay of <0.1 μs, which is small compared to the dwell time of the multi‐echo UTE sequence (1.7 μs).

Phantom and patient imaging highlighted the need for trajectory correction in UTE imaging, where uncorrected images were severely degraded. Characteristic UTE artifacts were previously described by Takizawa et al.^46^ These artifacts, namely halos and severe signal loss at boundaries and across the bulk of the subject, were substantially reduced by GSTF correction in our work. The second and third TE images exhibited shading artifacts for both corrected and uncorrected images, which were absent in UTE images, indicating that the UTE effectively mitigates off‐resonance effects seen at longer TEs. Potential sources of the shading artifacts are zeroth‐order effects such as B_0_ eddy currents, static B_0_ inhomogeneity (the Elekta Unity MR‐Linac lacks higher‐order shimming), or the vendor implementation of this sequence, where TEs are shifted up to 1 ms for some *k*_*z*_ partitions to accommodate variable *g*_*z*_ gradients.^23^ This variable‐duration slice encoding may also contribute to blurring along the slice direction in the UTE images.

Although this work focused on UTE imaging on the MR‐Linac, the GSTF correction technique can be applied to other imaging sequences that suffer from similar gradient distortions such as spiral imaging.^47^ In principle, any shape or combination of gradient waveforms can be corrected for gradient system imperfections without additional measurements. Moreover, GSTF‐based corrections may be performed once in advance so that the corrected trajectory is always available at reconstruction.

Broadly, there are two approaches to measuring the GSTF of a scanner: using specialized field probes or using simple‐phantom measurements.^8^, ^17^ Whereas field‐probe methods can be more precise, such detail is likely unnecessary for UTE imaging.^48^ One substantial advantage of phantom‐based approaches is their cost‐effectiveness and accessibility compared to specialized field probe equipment.^8^ The GSTF measurement method used in this study utilized a thin‐slice approach without in‐plane phase encoding,^15^ which can allow for a GSTF measurement up to the first order.^14^ Bruijnen et al. conducted a study similar to the present work,^45^ characterizing both zeroth‐ and first‐order GSTFs for UTE imaging on a 1.5 T Elekta Unity MR‐Linac. However, their measurement of the first‐order GSTF was performed with a single‐point imaging approach.^49^ In a separate study, Kronthaler et al. validated a thin‐slice method that used a chirp waveform instead of triangular pulses to measure the first‐order GSTF of a 3 T diagnostic system for high‐resolution UTE bone imaging.^19^ They demonstrated that measured GSTF‐based trajectory corrections can outperform corrections based on the analytic, vendor‐parameterized GSTF models. Compared to the current study, single‐point imaging and chirp‐waveform methods could be more challenging to implement with scanner software. Another difference is that we used a slice thickness of 3 mm, compared, for example, to 1.5 mm used by Kronthaler et al. Due to the lower gradient amplitudes and slew rates on the MR‐Linac system, the signal magnitude for the largest impulse is still approximately 70% in our measurement, allowing for accurate phase estimation.

One limitation of our work is that zeroth‐order effects were not measured where triangular‐pulse approaches allow for it.^17^ Although zeroth‐order effects are not expected to impact UTE imaging, they might impact the second and third TE images.^19^ However, comparing acquired images in Bruijnen et al.'s work,^45^ where Elekta Unity MR‐Linac UTE imaging was corrected using zeroth‐ and first‐order GSTFs, the images have a similar severity of shading artifacts suggesting that zeroth‐order corrections would not lead to substantial improvements. But sequences that do not rebalance phase or have long readouts, such as SSFP, EPI, or spiral imaging, would benefit from zeroth‐order corrections. A second limitation is that repeatability measurements were made only once, within a relatively short amount of time (48 h). Additional measurements may reveal greater degrees of day‐to‐day variation or longer‐term drifts. Furthermore, the GSTF could potentially be affected by the MR‐Linac gantry position or the temperature of gradient coils, which could increase due to scanning or environment. Thirdly, no comparisons were made to the trajectory corrected by the vendor's model because we did not have access to it for offline reconstruction. A fourth limitation is that no correction was made for receiver coil flare or geometric distortion caused by spatial gradient nonlinearity. Finally, the self‐navigation technique used in this work was based solely on the value of the respiratory surrogate and did not distinguish between inspiration and expiration.^28^, ^29^ Although this approach can characterize the extent of respiratory motion needed in adaptive MRgRT, it does not allow visualization of the hysteresis of the respiratory cycle. This contrasts with 4D CT, where 10 respiratory bins are typically used to sort a full breathing cycle, with motion states 1 and 10 representing end‐expiration.

## 5 CONCLUSION

A simple phantom‐based GSTF measurement and standard MR‐Linac scanner hardware (Elekta) were used to estimate *k*‐space trajectories for 4D UTE imaging to improve synCT generation methods of adaptive MRgRT for lung cancer. Corrected images were substantially improved from uncorrected images, exhibiting minimal halo artifacts and signal loss, and highlighting the need for trajectory correction in UTE imaging.

## FUNDING INFORMATION

u.o., p.n., and a.w. acknowledge funding from the Cancer Research UK program, grant C33589/A28284. b.l. is supported by the Cancer Research UK Convergence Science Centre at The Institute of Cancer Research (ICR), London, and Imperial College London, grant A26234.

## Supporting information


**Figure S1.** Uncorrected and corrected patient images. The 1st (end‐expiration) respiratory phases are shown at three echo times (UTE, TE_1_ = 0.18 ms, TE_2_ = 1.8 ms, and TE_3_ = 3.5 ms), acquired during free breathing on an MR‐Linac for patient 1 (A) and patient 2 (B). Multi‐echo images were reconstructed without and with gradient system transfer function (GSTF) correction. Difference images are shown. Note that UTE difference images are displayed with windowing five times larger than for the other echo times.
