# Supplementary material for: Gradient system characterization of a 1.5 T MR‐Linac with application to 4D UTE imaging for adaptive MR‐guided radiotherapy of lung cancer
Source: Magn Reson Med. 2025 Mar 19;94(1):28–40. doi: 10.1002/mrm.30505 (PMC12021324; doi:10.1002/mrm.30505)
Supplement: Supplementary file 1 — Figure S1. Uncorrected and corrected patient images. The 1st (end‐expiration) respiratory phases are shown at three echo times (UTE, TE1 = 0.18 ms, TE2 = 1.8 ms, and TE3 = 3.5 ms), acquired during free breathing on an MR‐Linac for patient 1 (A) and patient 2 (B). Multi‐echo images were reconstructed without and with gradient system transfer function (GSTF) correction. Difference images are shown. Note that UTE difference images are displayed with windowing five times larger than for the other echo times. [file MRM-94-28-s001.docx]

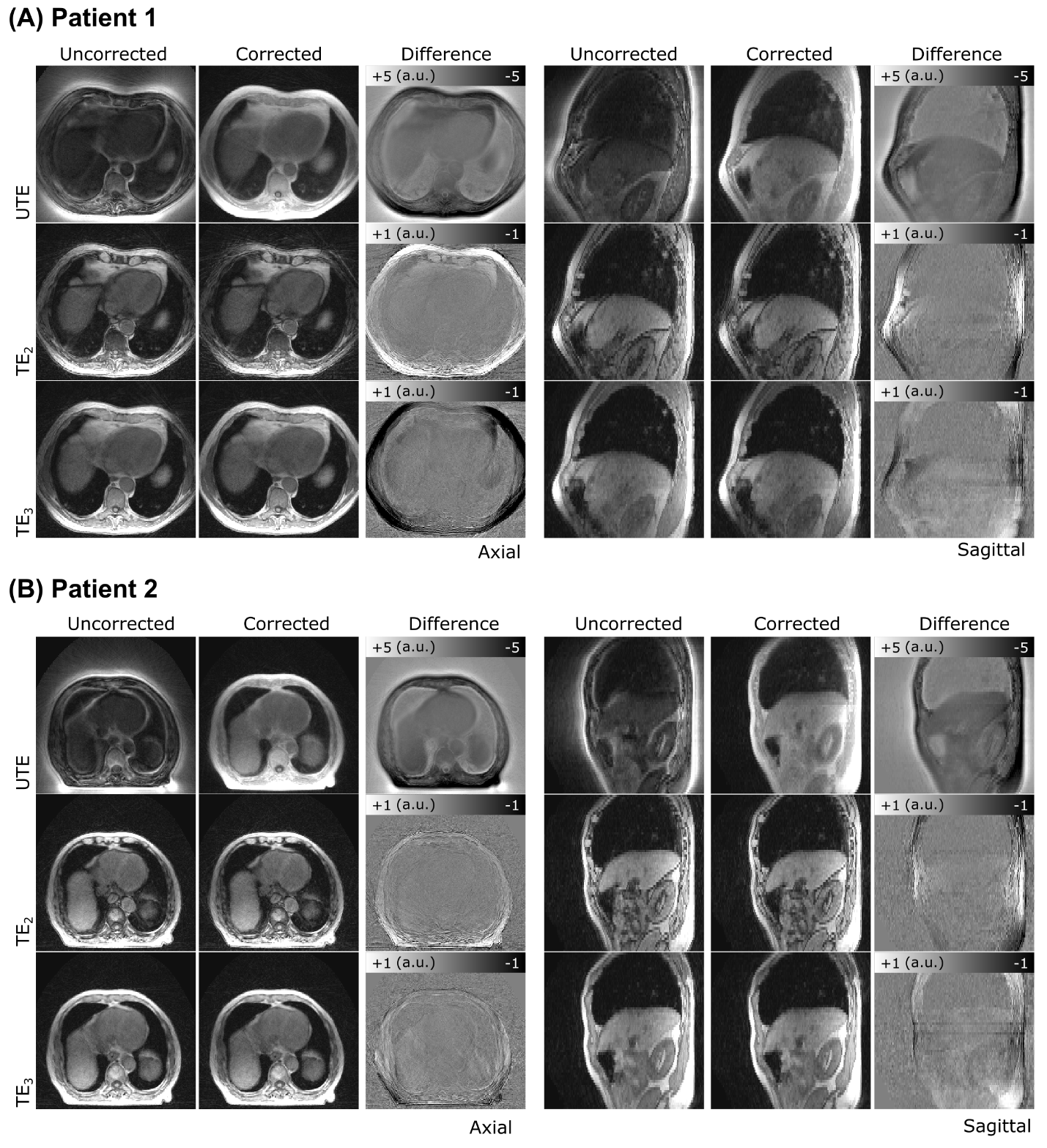


Figure S1: Uncorrected and corrected patient images. The 1st (end-expiration) respiratory phases are shown at three echo times (UTE, TE_1_ = 0.18 ms, TE_2_ = 1.8 ms, and TE_3_ = 3.5 ms), acquired during free breathing on an MR-Linac for patient 1 (A) and patient 2 (B). Multi-echo images were reconstructed without and with GSTF characterization of an MR-Linac for adaptive MR-guided lung treatment GSTF correction. Difference images are shown. Note that UTE difference images are displayed with windowing five times larger than for the other echo times
